# Ten Simple Rules for Getting Involved in Your Scientific Community

**DOI:** 10.1371/journal.pcbi.1002232

**Published:** 2011-10-27

**Authors:** Magali Michaut

**Affiliations:** The Donnelly Centre, University of Toronto, Toronto, Ontario, Canada

A scientific community consists of scientists working in a particular field of science and, most importantly, of their relationships and interactions. Beyond the traditional publication of research projects, discussions occurring during conferences, seminars, and even online through social networks or blogs enable ideas to spread more efficiently and are essential for building a lively and dynamic community. Activities such as organizing conferences and workshops, answering questions and discussing scientific ideas online, contributing to a scientific blog, or participating in open source software projects are typically thought of as outside classic research activity. Having scientists involved in those activities, however, is very important for the community to be dynamic and to promote fruitful discussions and collaborations. Scientific associations have an important role in enabling science by bringing people together and giving them a voice. Moreover, being involved in such activities is individually very rewarding because it enables scientists to acquire new skills not typically taught and to expand their network and interactions.

For those reasons, I encourage young scientists to get involved in their scientific community. However, it should be noted that this involvement takes time during which you are not directly contributing to your research projects and publications. It is thus essential to balance those activities. The purpose of this paper is twofold: i) illustrate some of the benefits of being involved and, most importantly, discuss how to get there; and ii) give some concrete advice and rules to keep this involvement as effective and controlled as possible in order to serve the community and receive benefits in return without hampering your research activity.

In scientific societies or associations, many tasks are accomplished by individuals who volunteer their time. Even tasks that appear to be merely administrative or clerical are essential for the scientific community and will make a difference in your field. In those volunteer organizations, projects are often driven by a single person or a very small team. Consequently, volunteers often have to take initiative and take things into their own hands. That is the context in which these rules should be of particular interest.

I have been involved in the Student Council of the International Society for Computational Biology for five years, progressively taking on more responsibilities, in particular in the organization of conferences (co-chair of the symposium in Boston in 2010 and chair of the first European symposium in Ghent in 2010), but also more generally in the Student Council (I was secretary—one of the elected leaders—of the Student Council in 2009). In addition, I created the French Regional Student Group (RSG-France), which I chaired for two years. This paper is based on my experience in the bioinformatics community, but also on associative involvement I had outside science. Most examples are taken from the bioinformatics community, but I believe the rules are rather general and apply for other communities.

## Rule 1: Collect Information

Maybe you are not sure whether you want to get involved or not and which kind of involvement is possible and would be interesting for you. The first thing to do is certainly to ask people around you about their experience in various associations and committees, should it be in your scientific community or other communities. You can ask them about the kind of involvement they have or had and what they like or dislike about it. Which were the benefits? Which were the problems? Would they do it again? All these questions can help you get a more concrete idea. In addition, you can search on the Internet and look for information about societies or associations you are interested in, if they exist. If they don't, it can also be good to create something new, but that is more challenging and may not be appropriate for a first experience.

## Rule 2: Define What You Want and Expect

It is important to know why you are getting involved and to define a clear goal. This will help you keep the motivation. For instance, you want to be part of a team of international students to improve your communication skills, or you want to learn how you can raise funds and contact sponsors. Maybe you want to get experience in organizing a conference or simply meet new colleagues all around the world. Defining what you will get or expect to get from the involvement is certainly a good idea. You might realize afterwards that you actually got very different benefits from what you were expecting, but it is good to think about it at first.

## Rule 3: Define Your Boundaries

To keep the balance between your activities you need to define clear boundaries, in particular to what extent you want to get involved. If you don't know what you are doing, you don't know when to stop. This is true for the daily work when you are wasting a lot of time simply because the task is not clear. But it is also valid for the duration of your involvement. It may be a good idea to decide beforehand when you want to stop. Do you plan to be involved two years? Three years? Until you get your PhD? Until you finish your postdoc or any other project? It may be easier to get involved after you have settled in your current place and project, as opposed to during phases of transition.

## Rule 4: Jump into the Pool and Get Involved

Now you want to get involved, you know why, and you have a goal and boundaries. But how can you actually start? Keep in mind that it may be enough to simply be open to any good opportunity that can unexpectedly happen. Haven't you been asked already to help out with the organization of an event or the reviewing of some abstracts? Otherwise, you will need to be proactive to get involved, and there are many ways to start. For instance, you can send an e-mail to a committee chair or the society chair asking questions about how it works or how you could help. You can even indicate your interest if you have some ideas or know what you would like to do, but it is certainly not required. Don't hesitate to contact people and just ask if there is anything you can do to help. Help is often needed and very appreciated. You can also attend the annual meeting of the society, join a committee, or participate in mailing list discussions. Even when you are already involved you can be proactive about taking on more responsibilities. If would like to do more, or change what you are working on, let people know and offer to do something different or new. It is always very motivating for the team to see that volunteers want more responsibilities.

## Rule 5: Let Other People Know What You Want to Do

Everybody has different interests and it is key to know them to build a team as effective as possible. If Joe hates contacting potential sponsors but likes writing meeting reports, he will be happy to know that William would rather be part of the fundraising effort and hates writing reports. Thus, be clear about your interests for the benefit of everybody. Following this idea, it is important to be clear with yourself and with others about what you can or can't do. You have to realize that you are part of a team. The point is not to do everything, or to take as many tasks or responsibilities as possible to show you are very much involved. The point is to commit to what you can do and to do it (and do it as well as you can). If you have some more time, you can always ask for more, help on other tasks, and get more involved. But if you can't deliver what you signed up for, you penalize the team and the work of other people. You can think of it as a soccer team—if you commit for a game and don't show up, the team is stuck.

## Rule 6: Dedicate Regular Time

It is extremely important to work regularly even when you are busy. It is indeed very likely that your research will take up all the time that is not firmly reserved for other activities. Thus, if you don't take your involvement as seriously as your research, you will never get anything done. When you feel overwhelmed, postponing everything for later when you expect to have more time is generally not a good strategy, because you will always be busy. It is often the case that 10 or 15 minutes on a project can be enough to get the next step done. Think about where you are and what is the next step. Maybe you just need to send an e-mail to ask about the quotes Jack had to get, or remind this keynote speaker about the picture he has to send. However, we still have some periods when it is more difficult than usual to dedicate the smallest amount of time. In that case, be clear about it and try to give your expected schedule and deadlines in advance so that other people on the team can adjust.

## Rule 7: Organize Your Time

Since you can't spend all your time on your community involvement and want to maintain a balance with the activities directly related to your research projects, it is essential to get organized. You can decide in advance how much time you want to dedicate and track the time you actually spend on your various activities. You might realize that some tasks take much more time than you were expecting or, conversely, are much faster to perform than you initially thought. The more you do it, the more accurate you become in your time estimates. This will enable you to know precisely which responsibilities and tasks you are able to handle and to be reliable in your commitments. As part of your schedule, you also want to define realistic milestones and deadlines, and stick to them.

## Rule 8: Work in a Team

Unless you are really working on a project alone, you will likely be part of a team and you should take advantage of it. Thus, don't take all the work for you, and remember that you are not alone. Keep in mind, particularly if you lead a team, that you need to distribute the work, delegate some tasks to others, and ask for help when you need it. In general it is good to assign a single responsible person and a deadline for each task. Working with other people is also an interesting way to get feedback on your work and ideas. Even though it usually takes more time, it is a good idea to suggest a discussion and take the opportunity to get comments on your ideas, actions, and concerns. That is what teamwork is about. Finally, this is probably more geared towards leaders, but it is extremely important to be able to get the best out of a group of different and complementary volunteers. Identify the strengths and weaknesses of your team workers and help everybody achieve their best based on their interests and skills. Identify and respect the differences of the people in the team. In particular, in international associations you will likely be interacting with people from all over the world who may have cultural differences in work styles, expectations, and ways to communicate. In line with this, it may be useful to provide an action item list with concrete tasks that allows people to find where they can help in the project.

## Rule 9: Encourage Others to Get Involved

Don't hesitate to let your colleagues know about your involvement. The point is not to show them how great you are doing and that they should do the same. But it is very likely that many people are not aware of this kind of involvement and don't realize how useful it is for the community and for you. Explain the work you are doing and what you get from it. You can encourage your colleagues to play an active role in the scientific community. If you think that someone would be effective in some specific task, tell him or her so. Sometimes people don't realize that they are good in specific tasks that seem complicated for others. For instance, you can ask Averell, who has very good graphical skills, to work on the design of various documents, flyers, or posters. Since the organization is composed of volunteers, it is often the case that people have to step down from their position when their job situation changes. Thus, it is important to have other volunteers who can take over. But it is also important to get new people to bring fresh ideas, new perspectives, and different ways to work. When you start to know people and have experience working with them, for example, in organizing a conference, you can be very effective doing similar tasks again. Nevertheless, it is rewarding to get new people involved and to have new comments from outside, even if it seems more complicated and takes more time. Last but not least, you should guide interested people to get involved. Many people would be happy to help but don't take the time to actually start, or don't feel confident enough. If you mentor them in the beginning, it might be enough for them to get into it.

## Rule 10: Enjoy as Much as Possible

What you like, you will do great without specific effort. If you know why you are doing it and if you enjoy it, you will take the time to do it, and you will do it well. And if you don't like it anymore or get bored, then finish your commitments and discontinue that activity. Of course, I should emphasize here that you have to finish your commitments first (see teamwork comments above)!

I hope I managed to illustrate that getting involved in your scientific community is not only extremely rewarding for you, but also possible for everybody, and that simple rules can help you balance your activities. There is a lot to do, various tasks for various people and at different levels of involvement. Every experience is of course different, and I would be glad to hear about your experience, should it be similar or very different. It is possible that you will have a bad experience or that something you try will not work out. In that case, don't be discouraged and try something else. Your experience can also simply be different from what you were expecting, but in the end, it is always a good experience. After all, experience is what you get when you didn't get what you wanted.

